# Deep brain stimulation for the treatment of Alzheimer’s disease: A safer and more effective strategy

**DOI:** 10.4103/NRR.NRR-D-24-01088

**Published:** 2025-06-19

**Authors:** Fan Zhang, Yao Meng, Wei Zhang

**Affiliations:** 1 Department of Neurology, Beijing Tiantan Hospital, Capital Medical University, Beijing, China; 2 Center for Cognitive Neurology, Department of Neurology, Beijing Tiantan Hospital, Capital Medical University, Beijing, China

**Keywords:** Alzheimer’s disease, amyloid-β, cholinergic system, deep brain stimulation, entorhinal cortex, fornix, hippocampus, mechanisms, nucleus basalis of Meynert, therapy

## Abstract

Alzheimer’s disease is the most common type of cognitive disorder, and there is an urgent need to develop more effective, targeted and safer therapies for patients with this condition. Deep brain stimulation is an invasive surgical treatment that modulates abnormal neural activity by implanting electrodes into specific brain areas followed by electrical stimulation. As an emerging therapeutic approach, deep brain stimulation shows significant promise as a potential new therapy for Alzheimer’s disease. Here, we review the potential mechanisms and therapeutic effects of deep brain stimulation in the treatment of Alzheimer’s disease based on existing clinical and basic research. In clinical studies, the most commonly targeted sites include the fornix, the nucleus basalis of Meynert, and the ventral capsule/ventral striatum. Basic research has found that the most frequently targeted areas include the fornix, nucleus basalis of Meynert, hippocampus, entorhinal cortex, and rostral intralaminar thalamic nucleus. All of these individual targets exhibit therapeutic potential for patients with Alzheimer’s disease and associated mechanisms of action have been investigated. Deep brain stimulation may exert therapeutic effects on Alzheimer’s disease through various mechanisms, including reducing the deposition of amyloid-β, activation of the cholinergic system, increasing the levels of neurotrophic factors, enhancing synaptic activity and plasticity, promoting neurogenesis, and improving glucose metabolism. Currently, clinical trials investigating deep brain stimulation for Alzheimer’s disease remain insufficient. In the future, it is essential to focus on translating preclinical mechanisms into clinical trials. Furthermore, consecutive follow-up studies are needed to evaluate the long-term safety and efficacy of deep brain stimulation for Alzheimer’s disease, including cognitive function, neuropsychiatric symptoms, quality of life and changes in Alzheimer’s disease biomarkers. Researchers must also prioritize the initiation of multi-center clinical trials of deep brain stimulation with large sample sizes and target earlier therapeutic windows, such as the prodromal and even the preclinical stages of Alzheimer’s disease. Adopting these approaches will permit the efficient exploration of more effective and safer deep brain stimulation therapies for patients with Alzheimer’s disease.

## Introduction

### Alzheimer’s disease

#### Epidemiological, pathological, and clinical characteristics

Alzheimer’s disease (AD), which accounts for 60%–80% of all patients with cognitive impairment, is the most common cause of cognitive disorders (Alzheimer’s Association Report, 2023). According to the 2023 report from Alzheimer’s Disease International, the number of dementia sufferers worldwide is projected to reach 139 million by 2050, and this compares to 55 million in 2019. Furthermore, the costs associated with dementia are expected to more than double, leading to significant economic and social challenges. With an aging population, AD imposes an increasingly heavy burden on patients, caregivers, families, and countries.

Pathologically, the typical features of AD include the formation of neuroinflammatory plaques outside neurons from amyloid-β (Aβ) and the development of neurofibrillary tangles inside neurons from hyperphosphorylated tau (P-tau) (Long et al., 2025; Zhang et al., 2025). These processes are then followed by neuronal degeneration and death (Zeng et al., 2025). These neuropathological hallmarks are widely recognized as core biomarkers for the diagnosis and staging of AD (Jack et al., 2024). Based on the pathological and clinical manifestations of AD, the concept of the AD continuum has been proposed; this concept describes the progression of AD from subtle brain alterations to more significant changes that result in clinical symptoms, such as memory decline and physical incapacity (Sperling et al., 2011).

Clinically, the main manifestations of AD are cognitive impairment, neuropsychiatric symptoms and impaired activities of daily living. According to the age of onset, AD is generally divided into early-onset AD (EOAD) and late-onset AD (LOAD). EOAD often manifests before the age of 65 years and makes up 5%–10% of all instances of AD (Zhu et al., 2015). Compared to LOAD, EOAD is more likely to involve gene mutations, with the most commonly mutated genes being amyloid precursor protein (APP), presenilin 1 (PSEN1) and presenilin 2 (PSEN2) (Dai et al., 2018). In the AD continuum, preclinical AD refers to the asymptomatic period during which related pathophysiological processes undergo progression (Sperling et al., 2011). The clinical staging of AD is applicable only to individuals within the pathophysiological continuum of AD. According to the revised criteria for diagnosis and staging of Alzheimer’s disease issued by Alzheimer’s Association workgroup in 2024, the clinical staging is defined as 7 stages (**Table 1**; Jack et al., 2024). The establishment of an AD continuum may facilitate early treatment in the preclinical and mild cognitive impairment stages of AD, and thus prevent or delay the dementia associated with AD.

**Table 1:** Clinical staging for individuals on the Alzheimer’s disease continuum

Clinical stage	Definition
Stage 0	Asymptomatic, biomarker negative, and deterministic gene only.
Stage 1	Asymptomatic, biomarker evidence only.
Stage 2	Mild detectable change with minimal impact on daily function.
Stage 3	Cognitive impairment with early functional impact.
Stage 4	Dementia with mild functional impairment.
Stage 5	Dementia with moderate functional impairment.
Stage 6	Dementia with severe functional impairment.

#### Therapeutic strategy

The current therapeutic approaches for AD are categorized into drug and non-drug treatments. Of the drug treatments, the most common medications available to alleviate the symptoms of AD include cholinesterase inhibitors and memantine, a N-methyl-D-aspartate (NMDA) receptor antagonist. Cholinesterase inhibitors increase the levels of acetylcholine in the brain, thereby reducing symptoms, while memantine additionally prevents neurodegeneration by inhibiting glutamatergic neurotoxicity, thus helping to reduce neuronal death (Khan et al., 2023; Tari et al., 2023). The U.S. Food and Drug Administration (FDA) has approved donepezil, rivastigmine, galantamine, memantine and the combination of memantine with donepezil as therapeutics for AD. However, these drugs primarily reduce symptoms and do not delay the progression of disease by targeting underlying pathological changes in the brain (2023). With regards to disease-modifying therapies, the FDA had approved aducanumab, lecanemab, and donanemab to clear Aβ from the brain and delay both cognitive and functional deterioration in patients with early stages of mild cognitive impairment (MCI) or mild dementia due to AD; however, these drugs cannot reverse or cure the disease (Budd Haeberlein et al., 2022; van Dyck et al., 2023). In China, oligomannate is the first approved new drug for the treatment of AD and targets the brain–gut axis. This drug improves cognition by suppressing dysbiosis of the gut microbiota and by alleviating neuroinflammation in the brain (Wang et al., 2019). Novel stem cell therapies, including mesenchymal stem cells (MSCs), embryonic stem cells (ESCs), induced pluripotent stem cells (iPSCs) and neural stem cells (NSCs), have the potential to differentiate into various cell types, thus allowing them to replace damaged cells in the brain. These innovative stem cell therapies may become promising and effective alternative approaches for AD (Hoang et al., 2022; Khan et al., 2023). Currently, the 2024 Alzheimer’s drug development pipeline indicates that there are 164 trials assessing 127 unique drugs for AD and that these trials target various pathological processes, including Aβ, tau, inflammation, neurotransmitter receptors, and synaptic plasticity (Cummings et al., 2024). This underscores the ongoing development of drug treatments for AD and highlights the urgent need for the development of more novel therapies in the future.

Non-drug treatments are often used to preserve or enhance cognitive function, facilitate activities of daily living, and improve quality of life. A recent longitudinal study showed that a healthy lifestyle, including nutritious diet, regular physical exercise, positive social interactions, active cognitive engagement, and abstaining from alcohol and smoking, was associated with slower memory decline in older adults, even among those carrying the apolipoprotein E (APOE) ε4 allele, which is linked to a higher risk of AD (Jia et al., 2023). Dietary interventions, such as the Mediterranean diet and ketogenic diet, have been suggested to slow cognitive decline, although their specific efficacy has yet to be fully investigated (Roy et al., 2021; Barnes et al., 2023). Multidisciplinary care, massage and touch therapy, and music therapy have been shown to be effective for neuropsychiatric symptoms in patients with AD (Watt et al., 2019). Above non-drug treatments have demonstrated a positive impact, at least to some extent, and more multi-faceted non-drug strategies should be pursued in the future to improve the clinical symptoms and quality of life for patients with AD.

Despite some advances in research and clinical trials for AD, current treatment options for patients remain limited, and many challenges persist. There is still a need to develop more effective, targeted and safer approaches for patients with AD.

### Deep brain stimulation

**Figure 1:**
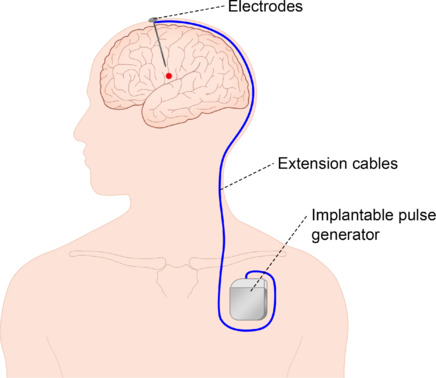
Diagram showing the DBS device. The DBS system consists of implanted electrodes within the brain, an IPG embedded under the chest, and extension cables running beneath the skin that connect the implanted electrodes to the IPG. The IPG delivers a continuous electrical signal to the brain at a predetermined frequency, pulse width, and amplitude. DBS: Deep brain stimulation; IPG: implantable pulse generator.

Deep brain stimulation (DBS) is an invasive surgical treatment that modulates abnormal neural activity by implanting electrodes into specific areas of the brain followed by electrical stimulation (Ohye et al., 1964). The DBS system consists of implanted electrodes within the brain and an implantable pulse generator (IPG) embedded under the chest. This system delivers a continuous electrical signal to the brain at a predetermined frequency, pulse width, and amplitude, with extension cables running beneath the skin to connect the implanted electrodes to the IPG (Miocinovic et al., 2013; **Figure 1**). The DBS system requires a specialized surgical setup to guide the electrode implantation process, which includes neuro-navigation techniques, real-time brain imaging and electrophysiologic monitoring. This advanced system allows patients or physicians to use an external controller to operate the pulse generator and adjust the parameters of electrical stimulation as needed.

**Figure 2:**
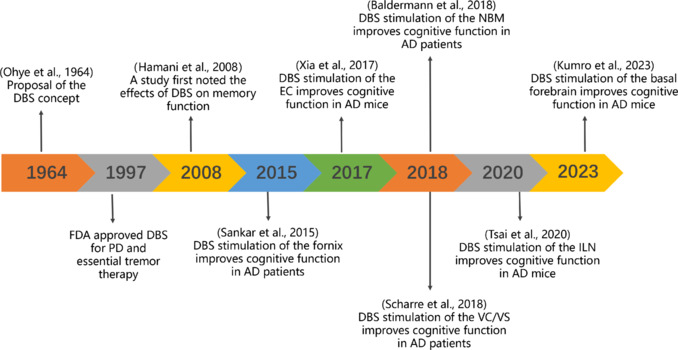
Timeline showing the application of DBS in research. The concept of DBS was proposed in 1964, and the FDA approved its use for treating PD and essential tremor in 1997. Research reported the effects of DBS on memory as early as 2008. Currently, a growing body of clinical and basic research has demonstrated the potential roles of DBS in treating cognitive disorders, particularly AD. AD: Alzheimer’s disease; DBS: deep brain stimulation; EC: entorhinal cortex; FDA: Food and Drug Administration; ILN: intralaminar thalamic nucleus; NBM: nucleus basalis of Meynert; PD: Parkinson’s disease; VC/VS: ventral capsule/ventral striatum.

DBS is currently used to treat a variety of diseases affecting the nervous system, including Parkinson’s disease (Sasikumar et al., 2023), epilepsy, obsessive-compulsive disorder, depression, and AD (Acevedo et al., 2023; Aiello et al., 2023; Cha et al., 2023). Hamani et al. (2008) investigated the effects of DBS on memory and reported that in a patient with morbid obesity, bilateral hypothalamic DBS evoked detailed autobiographical memory and increased recollection, but not familiarity-based recognition, thus indicating functional engagement of the hippocampus. Subsequent electroencephalographic source localization showed that hypothalamic DBS drove activity in the mesial temporal lobe, thus suggesting that electrical stimulation of the hypothalamus modulates limbic activity and improves hippocampus-dependent memory function (Hamani et al., 2008). A growing body of clinical and basic research clearly supports the potential of DBS for the treatment of cognitive disorders, particularly AD (Mao et al., 2018; Scharre et al., 2018; Jiang et al., 2022; **Figure 2**).

Furthermore, neurocomputational models have indicated that DBS significantly reduces the symptoms of AD and that the efficacy can be effectively controlled by appropriately adjusting key parameters, such as stimulation frequency, pulse width, current intensity, voltage, period, and duration (Yang et al., 2021). Intermittent stimulation refers to periods of stimulation followed by intervals of non-stimulation, while continuous stimulation involves uninterrupted stimulation. Both intermittent and continuous stimulation have been shown to influence the therapeutic effects of DBS (Koulousakis et al., 2019). As an emerging therapeutic approach, the efficacy of DBS for AD needs to be emphasized, and more in-depth studies should be conducted in the future.

Herein, we review research progress on the efficacy, underlying mechanisms, and limitations of DBS in clinical and basic studies for the treatment of AD. Our aim is to enhance understanding of the application of DBS in AD therapy and to identify new approaches for patients with AD.

## Search Strategy

We conducted a comprehensive literature search for AD and DBS over the past 10 years, up to April 2024, using PubMed. The search utilized the following keywords: “Alzheimer’s disease,” “deep brain stimulation,” “stimulation,” and “DBS.” We selected both basic studies and clinical trials that explored the effects or mechanisms of DBS on AD.

## Deep Brain Stimulation for Alzheimer’s Disease Treatment

**Figure 3:**
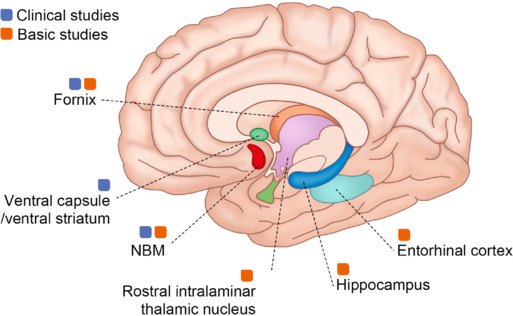
Target sites for deep brain stimulation in AD therapy. In clinical studies, the most commonly targeted sites include the fornix, NBM, and the VC/VS. In basic studies, the most frequently targeted sites include the fornix, NBM, hippocampus, entorhinal cortex, and rostral ILN. AD: Alzheimer’s disease; DBS: deep brain stimulation; ILN: intralaminar thalamic nucleus; NBM: nucleus basalis of Meynert; VC/VS: ventral capsule/ventral striatum.

There are various targets for DBS in AD therapy (**Figure 3**). In clinical studies (**Table 2**), the most common targets include the fornix, the nucleus basalis of Meynert (NBM), and the ventral capsule/ventral striatum (VC/VS). In basic research (**Table 3**), the frequently targeted sites are the fornix, the NBM, the hippocampus, the entorhinal cortex, and the rostral intralaminar thalamic nucleus (ILN). Each of these individual targets shows therapeutic potential and is associated with underlying mechanisms relevant to patients with AD (**Table 4**).

**Table 2:** Clinical studies of DBS for AD patients

Study	Stimulation target	Parameter setting	Outcome	Side effects
Sankar et al., 2015	Fornix	Undescribed	In some patients with AD, the volume of the hippocampus was found to be increased, accompanied by higher relative glucose metabolism in the hippocampus and a slower rate of atrophy in both the fornix and mammillary bodies.	Undescribed
Germann et al., 2021	Fornix	130 Hz, 90 µs, 1–10 V	Memory events were predicted with 72% accuracy by the fornix, anterior commissure, and the bed nucleus of the stria terminalis. The volume of tissue active during flashbacks was preferentially linked to a larger brain network, including the cingulate areas, prefrontal regions, medial and lateral temporal lobes, and insular cortex.	Undescribed
Lozano et al., 2016; Leoutsakos et al., 2018; Deeb et al., 2019	Fornix	130 Hz, 90 µs, 3–3.5 V	Increased cerebral glucose metabolism was observed in patients receiving stimulation after 6 mon, particularly in older adult participants (≥ 65 yr old). Both electrical stimulation and surgery were well tolerated and deemed safe.	A total of four acute serious device- or procedure-related safety events occurred in three patients with a rate of 7.1% of events/patient.
Rios et al., 2022	Fornix	130 Hz, 90 μs	Stimulating the Papez circuit and stria terminalis directly enhanced cognitive function, with the optimal stimulation site being at their point of direct interaction.	Undescribed
Lin et al., 2020	Fornix	130 Hz, 80 μs, 3 V	The patient's quality of life improved, as evidenced by a decrease in the ADL score from 65 to 47 points. Basic and instrumental functions improved, including dressing, eating, bathing, shopping, and trimming toenails, each showing a minimum 2-point drop in score. Additionally, the standardized uptake value ratio of 18F-FDG PET in brain regions associated with classical AD was elevated.	Undescribed
Mao et al., 2018	Fornix	130 Hz, 90 ms, 1–5 V	Three patients demonstrated improvements in mental state or social functioning, with one patient exhibiting exceptional enhancement in long-term memory.	No severe adverse events were observed.
Baldermann et al., 2018	NBM	5–20 Hz, 90–150 μs, 2–4.2 V	Preserved fronto-parieto-temporal cortical thickness was linked to better cognitive performance.	Undescribed
Kuhn et al., 2015b Kuhn et al., 2015a	NBM	20 Hz, 90 μs, 2.5 V	After 1 yr of stimulation, the ADAS-cog score deteriorated by an average of 3 points, while the mean MMSE score remained stable. Three out of four patients undergoing DBS exhibited a global increase in cortical glucose metabolism of 2%–5% on FDG-PET, with the most notable increases observed in the amygdalo-hippocampal and temporal areas. Two younger patients demonstrated that their MMSE and ADAS-cog scores remained largely stable.	Two serious adverse events occurred, both of which were hardware-related and resulted from malfunctioning plug-in connectors.
Scharre et al., 2018a	VC/VS	Undescribed	Compared to the ADNI group, all three individuals with AD demonstrated a lesser decline in their CDR-SB scale scores. The frontal cortical regions exhibited enhanced metabolism on FDG-PET.	No serious or permanent adverse events.

^18^F-FDG PET: Fluorine-18 fluorodeoxyglucose positron emission tomography; AD: Alzheimer’s disease; ADAS-cog: Alzheimer’s disease assessment scale-cognitive; ADL: activities of daily living; ADNI: Alzheimer’s disease neuroimaging initiative; CDR-SB: clinical dementia rating scale-sum of boxes; DBS: deep brain stimulation; FDG-PET: fluorodeoxyglucose positron emission tomography; MMSE: mini-mental state examination.

**Table 3:** Basic studies of DBS for patients with AD

Study	Stimulation target	Parameter setting	Outcome
Gondard et al., 2015b	Fornix	130 Hz, 2.5 V, 60 μs, 1 h	DBS promoted synaptic activity and electrical activity in the hippocampus, resulting in a dramatic elevation of cFos. In the forniceal region, DBS increased the levels of synaptic markers GAP-43, synaptophysin, and α-synuclein, as well as trophic factors such as VEGF and BDNF.
Gallino et al., 2019	Fornix	100 Hz, 3 V, 100 μs, 100 μA, 1 h	In the 3 and 6 wk following stimulation, DBS with electrode implants enhanced the performance of male mice in the Morris water maze. Compared to the sham group, DBS significantly increased the local volume of the right superior colliculus and decreased the local volume of the right central nucleus of the inferior colliculus
Leplus et al., 2019	Fornix	130 Hz, 80 μs, 100 μA, 5 wk	Forniceal DBS over 5 wk reduced amyloidosis, inflammatory response, and the loss of neurons in the hippocampal and cortical regions.
Huang et al., 2019	NBM	10 Hz, 50 Hz, 100 Hz, 130 Hz, 90 μs, 1 μA, 1 h/d	High frequency (100 Hz) for 21 d, starting at a young age (4 mon), was the ideal parameter for DBS. Under these conditions, soluble Aβ40 and Aβ42 in the cortex and hippocampus were significantly down-regulated. DBS decreased apoptotic cells and increased the number of surviving neurons in the hippocampal and cortical regions.
Koulousakis et al., 2019	NBM	Intermittent: 60 Hz,100 μs,200 μA, 20 s, on/40 s off Continuous: 20 Hz, 100 μs, 200 μA	Under bilateral-intermittent NBM-DBS, aged TgF344-AD rats performed better and maintained their performance longer in a spatial memory exercise compared to other conditions.
Kumro et al., 2023	Basal forebrain	60 Hz, 100 μs, 100 μA, 1 h/d for 5 mon	In the cerebral cortex of mice, long-term activation of the basal forebrain enhanced spatial memory, increased the expression of neurotrophin receptors, and decreased the levels of BACE1 and Aβ42.
Xia et al., 2017	EC	130 Hz, 90 μs, 1 h	In 6-wk-old mice, EC stimulation reversed early deficiencies in spatial memory and contextual fear, and subsequently reduced plaque load in Tg mice. In 6-mon-old mice, stimulation was similarly effective in alleviating memory problems in Tg mice. The DBS-induced improvement in memory appeared gradually over several weeks and was persistent and hippocampus-based.
Mann et al., 2018	EC	130 Hz, 90 μs, 50 μA, 7 h/d for 25 d	DBS in AD mice significantly reduced Aβ plaques in the frontal cortex and subiculum region, as well as the cellular level of Aβ42 in CA1. It also decreased P-tau in the cortex and T-tau in both the hippocampus and cortex, promoted neurogenesis in the dentate gyrus, and thereby improved hippocampus-dependent spatial memory deficits, restoring them to levels comparable to wild-type mice.
Akwa et al., 2018	EC	130 Hz, 90 μs, 50 μA, 1 h	DBS increased the level of synaptophysin in the CA1 region of AD mice. Synaptic activation promoted the clearance of Tau oligomers by autophagosomes and lysosomes, thereby reducing the level of Tau oligomers in the CA1 of AD mice.
Tsai et al., 2020	ILN	100 Hz, 500 μA, 60 μs, 30 min	DBS markedly enhanced performance in the Morris water maze and increased the expression of PSD-95 in the medial prefrontal cortex and hippocampus of rats injected with Aβ. It also maintained the densities of dendritic spines in the medial prefrontal cortex and pyramidal neurons in the hippocampus.

AD: Alzheimer’s disease; Aβ: amyloid-β; BACE1: beta-site amyloid precursor protein cleaving enzyme 1; BDNF: brain-derived neurotrophic factor; CA1: cornu ammonis 1; DBS: deep brain stimulation; EC: entorhinal cortex; GAP-43: growth-associated protein 43; ILN: intralaminar thalamic nucleus; NBM: nucleus basalis of Meynert; PSD-95: postsynaptic density protein 95; P-tau: hyperphosphorylated tau; T-tau: total tau; VEGF: vascular endothelial growth factor.

**Table 4:** Stimulation targets of DBS for patients with AD

Stimulation target	Anatomy and function
Fornix	The fornix is a C-shaped core white matter bundle in the limbic circuit, consisting of the head, crura, body, and columns. It connects the hippocampus with other brain regions in the limbic system, which is important for transferring complex cognitive information across the cerebral hemispheres.
Hippocampus and entorhinal cortex	The hippocampus is a curved and recurved sheet of cortex that folds into the medial surface of the temporal lobe. The entorhinal cortex, located anteriorly in the parahippocampal gyrus, and the hippocampus form a largely unidirectional network. The entorhinal cortex–hippocampus circuit is critical for memory formation, consolidation, and retrieval.
NBM	The NBM is a basal forebrain cholinergic nucleus. NBM-derived cortical cholinergic input is crucial for cognitive processing and attentional function.
VC/VS	The VC/VS is a significant neuroanatomical region located in the basal forebrain, connected to a network of brain regions involved in mood regulation and cognitive functioning.
ILN	The ILN is a collection of nuclei located within the internal medullary lamina of the thalamus, which is connected to the medial prefrontal cortex and contributes to various aspects of sensory and cognitive processing.

AD: Alzheimer’s disease; DBS: deep brain stimulation; ILN: intralaminar thalamic nucleus; NBM: nucleus basalis of Meynert; VC/VS: ventral capsule/ventral striatum.

### Fornix

#### Anatomy and function

The fornix is a C-shaped core white matter bundle in the limbic circuit, composed of four parts: the head, the crura, the body, and the columns. The fornix connects the hippocampus with other brain regions in the limbic system and represents an integral part of the classical Papez circuit, a major pathway in the limbic system that is primarily involved in memory. The fiber pathway of the fornix is divided into the pre-commissural and post-commissural fornix around the anterior commissure (Rudebeck et al., 2009). The fibers of the pre-commissural fornix primarily form the septohippocampal pathway, which is mainly cholinergic and has long been implicated in learning and memory (Dutar et al., 1995; Teles-Grilo Ruivo and Mellor, 2013). The post-commissural fornix comprises output fibers from the hippocampal subiculum to the anterior thalamic limbic nuclei, numerous hypothalamic nuclei and the mammillary bodies (Lavrador et al., 2019; Senova et al., 2020). The hippocampal subiculum is thought to be important for memory, including the processing of spatial, mnemonic, numeric, and movement information related to visual objects, as well as for stress responses (O’Mara, 2005).

#### Basic studies

A previous study involved male adult rats receiving 1 hour of DBS in the fornix. The stimulation was delivered at a frequency of 130 Hz, with a pulse width of 60 µs and a voltage of 2.5 V. These parameters promoted synaptic activity and electrical activity in the hippocampus, resulting in a significant elevation of cFos (Gondard et al., 2015). cFos is an immediate early gene that is rapidly and selectively regulated during hippocampal learning and memory processes (Rahmi et al., 2024). DBS in the fornix also increased the levels of brain-derived neurotrophic factor (BDNF) and vascular endothelial growth factor, both of which possess neurotrophic and neuroprotective potential beyond their pro-angiogenic activity. Furthermore, the levels of synaptic plasticity markers, including growth-associated protein 43, synaptophysin, and α-synuclein in the hippocampus, were significantly upregulated. These proteins are known to play major roles on axonal development and guidance, synaptic plasticity, synaptogenesis and memory processing. Modifications in the expression levels of these proteins may mediate the therapeutic benefits of DBS in the fornix (Gondard et al., 2015).

In another study, 2-month-old 3xTg mice were subjected to 1 hour of DBS at a frequency of 100 Hz, a pulse width of 100 µs, a voltage of 3 V, and a current of 100 µA in the fornix; analysis revealed that performance in the Morris water maze was significantly improved in male mice fitted with electrodes at 3 and 6 weeks post-DBS. In addition, DBS led to volume change trajectories in various brain areas. Compared to the non-DBS group, the DBS-treated group exhibited a significant increase in the volume of the right superior colliculus and a significant decrease in the volume of the right central nucleus of the inferior colliculus. The superior and inferior colliculi are associated with visual and auditory processing, respectively. These differences in visual and auditory processing may contribute to coordinated movements and affect performance in the Morris water maze test (Gallino et al., 2019).

In a study involving 18-month-old TgF344-AD rats administered with 5 weeks of DBS at a frequency of 130 Hz, a pulse width of 80 µs, and a current of 100 µA, it was observed a significant alleviation of inflammation, a reduced Aβ_42_ load, and reduced neuronal loss in both the hippocampus and the cortex. Furthermore, microglial and astroglial activations were reduced in AD mice after DBS. The above results may be associated with reduced inflammation, increased Aβ clearance and neuronal death in AD. These findings indicate the potential neuroprotective effects of DBS in the fornix (Leplus et al., 2019).

#### Clinical studies

Several clinical trials have investigated the use of DBS in the bilateral fornix of patients with AD. One study focused on patients with mild AD and investigated the acute flashback-like phenomena induced by DBS, specifically the involuntary recall of autobiographical memory. This trial involved the application of DBS via bilateral quadripolar leads (four contacts each) in the fornix, using a frequency of 130 Hz, a pulse width of 90 μs, and a voltage that started from a low level (approximately 1V) and was increased in 1V increment up to the maximum tolerable voltage (10 V). Analysis indicated that the fornix, the bed nucleus of the stria terminalis, and the anterior commissure were key sites for these flashbacks, achieving a 72% accuracy in predicting memory events. Furthermore, the volumes of brain regions activated during flashbacks were preferentially associated with a larger brain network, primarily involving the cingulate area, the prefrontal region, the medial and lateral temporal lobes and the insular cortex (Germann et al., 2021). In line with these findings, another study found that the majority of memory flashbacks (87%) were linked to dorsal brain contacts that likely stimulated both the fornix and the subcallosal regions (Deeb et al., 2019). Collecively, these results may provide valuable insights into the neuroanatomical basis of memory encoding and retrieval pathways.

In a prior study, patients with mild AD were randomly divided into DBS-on and DBS-off groups to receive either early DBS or sham stimulation, with delayed activation occurring after 12 months. The DBS-on group received continuous stimulation in the fornix (four contacts each) at a frequency of 130 Hz, a pulse width of 90 μs, and a voltage ranging from 3 to 3.5 V. Analysis demonstrated that cerebral glucose metabolism significantly decreased in the DBS-off group but increased in the DBS-on group in AD-related regions (temporal and parietal areas) as well as in relatively spared regions (sensory, motor, and cerebellar cortex). Notably, DBS significantly improved glucose metabolism in patients aged 65 years and older when compared to those younger than 65 years (Lozano et al., 2016). In addition, an investigation into the clinical efficacy and safety of sustained and delayed DBS over two years suggested a favorable safety profile for the treatment, with similar rates of adverse events observed in both trial phases (1 and 2 years). Consequently, DBS is considered a safe and effective treatment that can enhance cerebral glucose metabolism and produce potential benefits for older participants (≥ 65 years old). This indicates the need for further exploration of DBS as a therapeutic option for late-onset AD patients (Ponce et al., 2016; Leoutsakos et al., 2018).

A previous study performed quadripolar DBS in the fornix of patients with mild AD, using a frequency of 130 Hz, a pulse width of 90 μs, and a voltage ranging from 3.0 to 3.5 V. Analysis indicated that DBS stimulated neuronal activity within the memory circuit, including the entorhinal cortex and the hippocampus, while also activating the default mode network in the brain. Positron emission tomography (PET) scans revealed an early and significant reversal of impaired glucose utilization in the preserved temporal and parietal lobes. Further research demonstrated that patients with AD experienced a significantly slower rate of atrophy, with preserved or even increased bilateral hippocampal volumes following DBS in the fornix. Some patients exhibited improvements of cognitive function and/or a slowing of cognitive decline. These findings suggest that DBS has a circuit-wide effect by modifying neural circuit activity in neurodegenerative disorders, such as AD, thereby delaying the progression of brain atrophy and ultimately enhancing cognitive function (Sankar et al., 2015). In parallel, a longitudinal study treated mild AD patients with monopolar DBS in the fornix, using the same frequency of 130 Hz and a pulse width of 90 μs. This study revealed that stimulating the Papez circuit and the stria terminalis significantly improved cognitive performance, with the optimal stimulation site located at the lateral and posterior portions of the columns of the fornix (Rios et al., 2022).

In a recent case study, a 62-year-old male patient with severe AD underwent bilateral fornix DBS at 130 Hz, with a voltage of 3V and a pulse width of 80 μs. The patient’s quality of life improved significantly, and ^18^F-fluoro-2-deoxy-D-glucose (^18^F-FDG) scan indicated a significant increase in glucose metabolism in brain regions associated with attention deficit (Lin et al., 2020). Hence, DBS may enhance daily functionality by enhancing glucose metabolism in the brain.

Another study investigated quadripolar DBS in the bilateral fornix of patients with severe AD. The stimulation protocol involved a frequency of 130 Hz, a pulse width of 90 ms, and a gradual increase of voltage from 1 to 5 V. Analysis indicated that cognitive function improved in most patients, with the most significant enhancements observed in the scores of the Mini-Mental State Examination (MMSE) and the Montreal Cognitive Assessment scales. No serious neurological adverse events were reported. This study preliminarily verified the feasibility of DBS for the treatment of severe AD in terms of safety and efficacy, thereby enriching our current understanding of DBS for addressing various stages of AD (Mao et al., 2018).

According to these studies, DBS stimulation in the fornix can safely and effectively activate the neural circuits responsible for both memory and retrieval. This stimulation has been shown to increase brain glucose metabolism, slow the rate of brain atrophy, and ultimately improve cognitive function, thereby enhancing the quality of life for patients with AD.

### Nucleus basalis of Meynert

#### Anatomy and function

The nucleus basalis of Meynert (NBM) is a cholinergic nucleus located in the basal forebrain that influences both neocortical and subcortical structures by producing acetylcholine. The NBM is involved in all cortical networks, acts on receptors in glial cells, stabilizes cerebral blood flow, and stimulates the release of nerve growth factors (NGF). Cholinergic input from the NBM to the cortex is critical for cognitive processing and attentional function. Furthermore, neuronal abnormalities in the NBM are significantly associated with damage to cortical cholinergic neurons and the severity of cognitive impairment in patients with AD (Kumbhare et al., 2018).

#### Basic studies

A recent study investigated the effects of stimulation frequency, initiation timing, and duration of DBS in the NBM using an amyloid precursor protein/presenilin1 mouse model of AD. Analysis indicated that the optimal parameters for DBS were a high frequency of 100 Hz applied for 21 days, starting at an early age (4 months) (Huang et al., 2019). Under these conditions, levels of soluble Aβ_40_ and Aβ_42_ in the hippocampus and the cortex declined significantly. This decline may have occurred through the activation of the phosphatidylinositol 3′-kinase/Akt pathway and the inhibition of the extracellular signal-regulated kinase 1/2 pathway. Furthermore, oxidative stress was mitigated, cell apoptosis was reduced, and neuronal survival increased (Huang et al., 2019). DBS in the NBM also reduced the activity of acetylcholinesterase while increasing the activity of choline acetyltransferase in the hippocampus and the cortex of AD mice. Collectively, these changes contributed to an increase in acetylcholine level within the activated cholinergic system. As a result of these mechanisms, cognitive function improved in AD mice, as determined by the Morris water maze (Huang et al., 2019).

An earlier study that explored the effects of intermittent versus continuous DBS in the NBM using TgF344-AD rats found that both bilateral and unilateral intermittent DBS at a frequency of 100 Hz, a pulse width of 100 µs, a current of 200 µA, and a stimulation pattern of 20 seconds on followed by 40 seconds off, significantly improved cognitive performance in spatial memory tasks when compared to continuous DBS at 20 Hz (Koulousakis et al., 2019). Collectively, these results suggest that intermittent stimulation may be a more effective approach for DBS in AD.

Given that the NBM is a crucial component of the basal forebrain, a study was conducted to investigate the effects of stimulating the basal forebrain on AD. In this study, both AD mice and wild-type mice received DBS at a frequency of 60 Hz, a pulse width of 100 µs, and a current of 100 µA. Analysis showed that the DBS group performed significantly better in the Morris water maze test when compared to AD mice that did not receive stimulation. Furthermore, the DBS group exhibited increased expression levels of neurotrophin receptors, specifically tropomyosin receptor kinase A (TrkA) and TrkB, which are associated with NGF and BDNF in the frontal cortex. Furthermore, DBS led to a significant reduction in the expression of β-site amyloid precursor protein cleaving enzyme 1 (BACE1), the major β-secretase responsible for generating Aβ, and reduced Aβ_42_ accumulation in the cerebral cortex (Kumro et al., 2023). This effect was attributed to the activation of the basal forebrain, which releases acetylcholine and subsequently increases neurotrophin and neurotrophin receptor expressions while reduces the expression of BACE1 and the accumulation of Aβ (Wan et al., 2024). Collectively, these findings provide valuable insights into the relationship between basal forebrain stimulation and the activation of neurotrophin receptors, thus highlighting their role in reducing the generation and accumulation of Aβ_42_, and thereby providing a potential treatment for AD (Kumro et al., 2023).

#### Clinical studies

Bilateral stimulation of the NBM has been used as a target for DBS in several clinical trials. In a sham-controlled study, patients with mild to moderate AD were treated with monopolar DBS at a frequency of 20 Hz, a voltage of 2.5 V, and a pulse width of 90 µs. Collectively, these results indicated that cortical glucose metabolism increased by 2%–5% in three patients evaluated using ^18^F-FDG, with the most significant increases observed in the amygdalo-hippocampal and the temporal regions. The Alzheimer’s Disease Assessment Scale-Cognitive (ADAS-cog) score declined by an average of three points following a year of DBS, suggesting the relatively mild progression of AD, which was further supported by an increase in MMSE score. Additional investigations in two of the six patients, who were younger and less affected, showed that their MMSE and ADAS-cog scores remained relatively stable, thus suggesting that DBS may delay disease onset and improve cognitive performance. Thus, targeting the NBM with DBS could enhance cortical glucose metabolism and stabilize or slightly improve the symptoms of patients with AD without any significant stimulation-induced adverse effects (Kuhn et al., 2015a, b).

A follow-up study treated patients at advanced stage of AD with continuous DBS at a frequency of 20 Hz, a voltage of 2.0–3.0 V, and a pulse width of 90 µs. Under these conditions, the connectivity between the hippocampal network and the frontoparietal network was significantly strengthened. Moreover, the connectivity between the parahippocampal gyrus and the parietal cortex was also significantly increased. Furthermore, the proportion of parahippocampal gyrus -related connections was increased by the dynamic functional network analysis. The MMSE scores of AD patients significantly increased after one month of follow-up. Collectively, these findings suggest that DBS in the NBM enhances the connectivity of multiple cortical regions and large-scale brain networks, identifying this strategy as a promising therapeutic approach for AD (Jiang et al., 2022).

A previous study investigated the effects of quadrupolar DBS on patients with AD, utilizing frequencies ranging from 5 to 20 Hz, voltages from 2.0 to 4.2 V, and pulse widths between 90 and 150 μs. The aim of this particular study was to identify the degrees of atrophy in specific brain regions that could serve as predictors for the efficacy of DBS in the NBM. Analysis suggested that the beneficial effects of DBS were contingent upon the integrity of the front-parietal-temporal network. DBS in the NBM appeared to induce plasticity through direct or indirect interactions with this network, potentially benefiting patients who exhibited less severe atrophy prior to DBS treatment (Baldermann et al., 2018).

Overall, these investigations indicate that targeting the NBM with DBS can activate the cholinergic system, elevate levels of neurotrophic factors, increase cortical glucose metabolism, reduce Aβ_42_ accumulation, and improve cognitive performance. Notably, these effects depend on the integrity of the front-parietal-temporal interplay.

### Hippocampus and entorhinal cortex

#### Anatomy and function

The entorhinal cortex, located anteriorly in the parahippocampal gyrus, and the hippocampus form a primarily unidirectional network, with the entorhinal cortex providing input to the hippocampus and the fornix serving as the primary output structure. The entorhinal cortex is divided into two groups based on cytoarchitecture and connectivity: the medial group, which comprises spatial neurons, and the lateral group, which contains neurons that encode object information, attention, and motivation (Curot et al., 2017). The hippocampus is a curved and folded sheet of cortex that lies along the medial surface of the temporal lobe. It is divided into two primary subdivisions: the hippocampus itself and the parahippocampal gyrus. The hippocampus includes the dentate gyrus, cornu ammonis (CA) and the subiculum, and is tightly linked to the entorhinal cortex. Projections from the entorhinal cortex to the hippocampus follow two pathways, originating from the second and third cortical layers of the entorhinal cortex and terminating in the dentate gyrus and CA1, respectively.

An abundance of information exchange has been detected between the entorhinal cortex and other brain regions, as the entorhinal cortex can receive, process, and output data from these regions. Sensory afferent information gathers on the surface of the entorhinal cortex, where the second and third cortical layers transmit this information to sub-regions of the hippocampus. After processing the afferent information, the CA1 and subiculum transmit the output information back to the fourth and fifth layers of the entorhinal cortex, which then widely disseminates this output information to the forebrain cortex and subcortical areas.

Research has demonstrated that both the entorhinal cortex and the hippocampus play crucial roles on memory formation, consolidation and retrieval (Kosel et al., 1982; Squire et al., 2004; Masurkar et al., 2017).

#### Basic studies

An earlier study demonstrated that DBS in the CA1 region and the entorhinal cortex could reverse memory deficit in a scopolamine-induced rat model (Hescham et al., 2015). However, there is currently a lack of basic research focusing on hippocampal targets for DBS in AD. Nevertheless, other stimulation modalities targeting the hippocampus have shown efficacy, thus suggesting that DBS may be a promising and more direct stimulation approach for hippocampus-targeted AD therapy. For instance, in 3-month-old 5xFAD mice, a 4-week regimen of 40 Hz intracranial alternating current stimulation applied to the hippocampus as well as the subventricular zone significantly enhanced neurogenesis, as indicated by increased levels of Ki67, Nestin, and doublecortin when compared to sham stimulation. The neurogenesis achieved was even comparable to that seen in age-matched wild-type controls, indicating that intracranial alternating current stimulation could significantly enhance brain neurogenesis when used appropriately, thus presenting a promising avenue for the treatment of AD (Liu et al., 2020).

Another study found that DBS in the entorhinal cortex, using a frequency of 130 Hz, a pulse width of 90 µs, and a current of 0–500 µA for 30–120 minutes in 8-week-old wild-type mice, transiently stimulated proliferation in the dentate gyrus, leading to differentiation into neurons. These stimulation-induced neurons integrated into the hippocampal circuitry, supporting water-maze memory once they had matured sufficiently. Consequently, after 6 weeks, there was a significant improvement in water-maze memory (Stone et al., 2011). In 6-week-old AD mice, DBS in the entorhinal cortex with the same parameters (130 Hz frequency, 90 µs pulse width, and 1 hour of stimulation) significantly reduced Aβ plaque load in both the hippocampus and the cortex, restored spatial memory, and alleviated early contextual fear. This stimulation significantly rescued memory deficit in 6-month-old AD mice but did not reduce Aβ plaque load in these older mice. These data suggest that DBS may only exert its effects at the early stages of AD through a plaque-dependent mechanism, and improve memory via other pathways at the late stages of AD. Furthermore, the memory enhancements induced by DBS appeared to develop gradually over several weeks and were sustained, indicating a hippocampus-based mechanism (Xia et al., 2017). Supporting this, another study reported that DBS in the entorhinal cortex using a frequency of 130 Hz, a pulse width of 90 µs, and a current of 50 µA for 25 consecutive days, with 7 hours of stimulation per day, led to significant reduction in Aβ plaque in the frontal cortex and the subiculum, as well as a significant decrease in cellular Aβ_42_ level in the CA1. Furthermore, reductions of P-tau in the cortex and T-tau in both the hippocampus and the cortex were observed. Neurogenesis in the dentate gyrus was also promoted, which improved hippocampus-dependent spatial memory deficit to the level comparable to those of wild-type mice. Notably, the positive effects of DBS on recognition memory in AD mice persisted for at least one month after stimulation ceased, thus suggesting that DBS induced long-term alterations in brain function (Mann et al., 2018). In another investigation, DBS in the entorhinal cortex with the same stimulation parameters (130 Hz frequency, 90 µs pulse width, and 50 µA current for 1 hour) significantly upregulated synaptophysin level in the CA1 region of AD mice. This synaptic activation facilitated the clearance of Tau oligomer via autophagosomes and lysosomes, thereby reducing the level of Tau oligomer in the CA1 of AD mice (Akwa et al., 2018). Collectively, these findings imply that synapse preservation and the autophagic-lysosomal degradation of pathological Tau may be two protective mechanisms through which DBS operates in the entorhinal cortex.

#### Clinical studies

Early clinical research indicated that DBS in the entorhinal cortex, using a frequency of 50 Hz, a pulse width of 300 μs, and a current ranging from 0.5 to 1.5 mA, significantly enhanced spatial memory and reset the theta rhythm, as evidenced by electroencephalogram readings from the hippocampus in patients with epilepsy (Dlouhy and Rao, 2012). These findings suggest that the entorhinal cortex could be a viable target for DBS to improve cognitive function. Furthermore, this research supports the potential for enhanced cognitive function through DBS targeting the hippocampus and the entorhinal cortex. However, there remains a lack of sufficient studies specifically investigating DBS in the hippocampus and the entorhinal cortex, thus indicating a need for further exploration in this area.

### Ventral capsule/ventral striatum

#### Anatomy and function

The ventral caudate/ventral striatum (VC/VS) is an important neuroanatomical region located at the basal forebrain and represents an extension of the ventral striatum and includes the nucleus accumbens (Hitti et al., 2021). The nucleus accumbens is divided into two parts: the shell and the core, both of which play crucial roles in various aspects of reward processing, motivation, and reinforcement learning. The VC/VS is interconnected with a network of brain regions involved in cognitive function and mood regulation and receives inputs from the prefrontal cortex, which plays a critical role in executive functions, such as decision-making and impulse control. In addition, the VC/VS has connections to the hippocampus, which is vital for memory formation, and the amygdala, which is central to the processing of emotions, particularly fear and anxiety (Park et al., 2019).

#### Clinical studies

In a non-randomized controlled trial, patients with AD were treated with continuous DBS in the VC/VS, positioned 5 to 10 mm lateral to the midline, 2 to 4 mm anterior to the anterior commissure, and 2 to 5 mm ventral to the anterior commissure, for at least 18 months. This study aimed to optimize the frontal network and improve cognitive function. Analysis indicated that the decline in score on the Clinical Dementia Rating Scale- Sum of Boxes was significantly mitigated in the DBS group when compared to the control group. This was the first study to demonstrate the efficacy and safety of DBS targeting the VC/VS in patients with AD (Scharre et al., 2018). Currently, there is a lack of foundational studies focusing on the VC/VS in DBS. As an uncommon stimulation site, the VC/VS deserves greater attention in both basic and clinical research on DBS in the future.

### Intralaminar thalamic nucleus

#### Anatomy and function

The ILN are a collection of nuclei located within the internal medullary lamina of the thalamus that are connected to the medial prefrontal cortex and play a role in various aspects of cognitive processing (Phillips et al., 2021).

#### Basic studies

A previous study reported that DBS in rostral ILN at a frequency of 100 Hz, with a pulse width of 60 µs and a current of 500 µA, significantly enhanced performance in the Morris water maze of rats injected with Aβ. This stimulation also increased the expression of postsynaptic density protein 95 in the medial prefrontal cortex and the hippocampus of rats injected with Aβ, while maintaining dendritic spine densities in the medial prefrontal cortex and pyramidal neurons in the hippocampus (Tsai et al., 2020). Furthermore, there were no significant differences between the rostral ILN-DBS treatment group and the non-AD control group in terms of Morris water maze performance, the levels of postsynaptic density protein 95 in the medial prefrontal cortex and the hippocampus, or dendritic spine density in the medial prefrontal cortex and pyramidal neurons in the hippocampus. These data indicated that DBS contributed to the complete amelioration of Aβ-induced spatial memory impairment and dendritic regression. These results imply that the ILN plays a crucial role in neuronal plasticity and spatial memory function by exerting broad effects across several brain regions, thus providing a rationale for exploring the therapeutic efficacy of ILN-DBS in AD patients (Tsai et al., 2020). Currently, there is a lack of clinical studies targeting the ILN in DBS. These findings also support further investigations of the therapeutic efficacy of ILN-DBS in AD patients.

## Mechanisms of Deep Brain Stimulation in Alzheimer’s Disease Therapy

### Deep brain stimulation reduces the deposition of amyloid-beta

As an important pathological biomarker of AD, the excessive accumulation of Aβ triggers other pivotal pathological processes and leads to both neuronal degeneration and death. Amyloid plaques formed by Aβ aggregation can induce inflammatory responses around neurons and exacerbate neuronal damage through direct toxic effects, ultimately resulting in neuronal death and synaptic loss. Clearing Aβ has been shown to slow the progression of AD. Previous research demonstrated that DBS in the basal forebrain, the fornix, the NBM, and the entorhinal cortex of AD mice or rats reduces Aβ accumulation in both the hippocampus and the cortex (Xia et al., 2017; Huang et al., 2019; Leplus et al., 2019; Kumro et al., 2023; Wan et al., 2024). Collectively, these findings provide evidence that DBS can mitigate AD pathology.

### Deep brain stimulation activates the cholinergic system

The cholinergic neurons in the basal forebrain constitute the basal forebrain cholinergic system, which serves as the primary source of cholinergic projections to the prefrontal cortex, the hippocampus, the entorhinal cortex and the amygdala. The hippocampus and the entorhinal cortex receive direct input from this cholinergic system, and the release of acetylcholine in the hippocampus is associated with long-term potentiation and depression, which are critical for learning and memory. Therefore, the loss of cholinergic neurons and the depletion of acetylcholine underlie the impairments of above cognitive domains of AD (Berry and Harrison, 2023). As a key component of the basal forebrain, the NBM is a common target for DBS in the treatment of AD due to its role in regulating the cholinergic system. Currently, acetylcholinesterase inhibitors are used to alleviate symptoms in patients with AD, while the use of DBS to target the cholinergic system shows significant potential for treatment. Research has indicated that DBS in the basal forebrain and the NBM promotes the release of acetylcholine, increases acetylcholine levels in the activated cholinergic system, and enhances cognitive performance in a mouse model of AD (Huang et al., 2019; Kumro et al., 2023). Collectively, these findings provide a strong rationale for applying cholinergic stimulation through DBS in AD patients.

### Deep brain stimulation elevates the levels of neurotrophic factors

Neurotrophic factors are a family of peptides and small proteins that are essential for the growth, survival, and maintenance of neurons; these proteins are believed to play significant roles in the pathogenesis of AD. Of these factors, BDNF and NGF are particularly important in the context of AD (Wei et al., 2024). BDNF exhibits neuroprotective effects by mitigating Aβ-induced neurodegeneration, reducing synaptic abnormalities, and preserving neuronal viability, thereby helping to suppress cognitive impairment (Mitroshina et al., 2020). Furthermore, NGF enhances synaptic plasticity, promotes glutamatergic neurotransmission, diminishes inflammatory responses triggered by Aβ, and facilitates the repair of Aβ-induced synaptic deficit and loss by activating microglia (Rizzi et al., 2018). A previous study involving mice found that neurons within the basal forebrain cholinergic system were highly dependent on NGF during development and that a deficit in NGF might contribute to the impairment of this system, ultimately disrupting cognitive function (Berry and Harrison, 2023). BDNF and NGF typically bind to their respective receptors, TrkB and TrkA. Notably, DBS in the basal forebrain of AD mice has been shown to increase the expression of both TrkB and TrkA, which correlates with reduced Aβ accumulation and improved spatial memory (Kumro et al., 2023). Furthermore, DBS in the fornix of adult rats has been found to elevate the levels of vascular endothelial growth factor and BDNF (Gondard et al., 2015). Therefore, DBS may alleviate symptoms of patients with AD via the neuroprotective actions of various neurotrophic factors, thus enhancing neuronal health and cognitive function.

### Deep brain stimulation increases synaptic activity and plasticity

Synapses are the specialized intercellular junctions through which neurons connect and communicate. The intensity of synaptic transmission can be dynamically and permanently adjusted in response to variations in neuronal activity, a phenomenon known as synaptic plasticity. This capacity for synapses to alter their strength and structure in response to neural activity is crucial for learning and memory formation (Brown et al., 2022). Previous studies have identified that synaptic dysfunction is characterized by the loss of synapses, the enhancement of long-term depression, and the inhibition of long-term potentiation (Tampellini, 2015; Jang and Chung, 2016). These dysfunctions represent early events in the progression of AD and are strongly linked to cognitive decline. Research has shown that rats treated with DBS in the fornix and the entorhinal cortex exhibited improved synaptic plasticity, synaptogenesis and memory processing (Gondard et al., 2015a; Akwa et al., 2018). In addition to an increased density of synapses, the effects of high- and low-frequency DBS are believed to induce short- and long-term changes in synaptic efficiency. For instance, rats receiving long-term and high-frequency DBS in the fornix demonstrated significant long-term potentiation in the hippocampus (Hao et al., 2021). Thus, enhanced synaptic activity and plasticity may contribute to the therapeutic effects of DBS for patients with AD.

### Deep brain stimulation promotes neurogenesis

Neurogenesis is the process by which new functioning neurons are generated. This complex process begins with the division of neural stem cells, which can differentiate into neural progenitor cells that migrate and ultimately develop into new neurons (Amanollahi et al., 2023). In mammals, neurogenesis primarily occurs in two key regions: the subventricular zone along the outer walls of the lateral ventricles and the sub-granular zone of the dentate gyrus within the hippocampus. These areas contain multipotent and self-renewing neural stem cells that are essential for adult neurogenesis (Kim et al., 2022). Interestingly, emerging evidence suggests that the major molecular players involved in the regulation of AD also influence the generation of new hippocampal neurons. Notably, modifications in neurogenesis can occur prior to the development of AD pathological biomarkers or significant neuronal loss, thus indicating that alterations in neurogenic processes may serve as early indicators of the disease (Mu and Gage, 2011).

Previous studies have also demonstrated that DBS in the fornix and entorhinal cortex restored or promoted neurogenesis in AD mice and enhanced spatial learning and memory (Hao et al., 2015; Ronaghi et al., 2019). Collectively, these findings highlight the importance of neurogenesis in the treatment of AD through DBS.

### Deep brain stimulation enhances glucose metabolism

The human brain has the highest rate of energy metabolism in the body. Despite accounting for approximately 2% of total body weight, the adult brain receives approximately 15% of cardiac output while awake and at rest, utilizing almost 20% of total body oxygen consumption and 25% of total body glucose. Glucose is the primary and essential energy source for the brain (Chen and Zhong, 2013). Energy consumption in the brain is primarily derived from glucose metabolism, which consists of two main processes: intracellular oxidative catabolism and glucose transport. Due to its high energy demands, the brain is particularly susceptible to impaired energy metabolism (Minoshima et al., 1997). A growing body of research has demonstrated a direct correlation between the onset and progression of AD and dysfunction in glucose metabolism in the brain (Pagani et al., 2017; An et al., 2018; Johnson et al., 2020). One study indicated that a gradual decline in glucose metabolism in the brain, as measured by FDG-PET, may serve as a marker for mild cognitive impairment due to AD (Pagani et al., 2017). In an experimental study involving rats, DBS in the fornix was found to increase glucose metabolism in the medial limbic and the corticolimbic circuits, while decreasing glucose metabolism in the primary motor cortex, the primary somatosensory cortex, the primary visual cortex and the cerebellum (Shin et al., 2019). Clinical trials have shown that DBS in the fornix and the NBM in AD patients increased cortical glucose metabolism and slightly enhanced cognitive function (Laxton et al., 2010; Smith et al., 2012), thus providing evidence for the role of glucose metabolism in the treatment of AD with DBS.

## Limitations

As an invasive treatment, DBS has been associated with several adverse effects in clinical trials. Common complications related to DBS surgery include symptomatic hemorrhage, wound infection and hardware failure. General complications may also include seizures, delirium or psychosis, venous air embolism, ischemic stroke, nosocomial infections, contact dermatitis, headache, nausea, and various other medical issues, including falls, muscle spasms, urinary tract infection, urgency, incontinence, upper respiratory infection and dyspnea (Picton et al., 2024). Serious adverse effects of DBS in patients with AD are rare. An incidence of 7.8% for all adverse effects was reported (Ponce et al., 2016). There appear to be no long-term complications of DBS, and the frequency of adverse effects tends to decline over time. With regards to patient selection criteria, current DBS treatments for AD primarily focus on individuals with mild-to-moderate AD, where efficacy has been observed to be greater. Notably, DBS significantly improved glucose metabolism in patients aged 65 years and older when compared to those younger than 65 years, thus supporting its potential as a treatment for patients with late-onset AD (Lozano et al., 2016). However, there is currently insufficient long-term follow-up data on AD patients treated with DBS, leaving the long-term effect of this intervention unclear. Future studies with larger populations are necessary to clarify the long-term efficacy of DBS and its associated adverse events. Other neuromodulation techniques, including transcranial magnetic stimulation and transcranial direct current stimulation, are emerging as potential treatments for AD. These methods have been shown to improve the clinical symptoms of AD (Pople et al., 2020). While DBS is invasive, it offers more precise and direct stimulation of brain targets compared to other neuromodulation techniques. No previous studies have attempted to compare the effects of these neuromodulation therapies, thus highlighting the need for further investigation into the efficacy of DBS relative to other neuromodulation techniques for AD (**Table 5**).

**Table 5:** Comparison of different neuromodulation techniques

Technique	Deep brain stimulation	Transcranial magnetic stimulation	Transcranial direct current stimulation
Invasiveness	Invasive	Non-invasive	Non-invasive
Principle	Delivering continuous or periodic electrical stimulation to neurons by implanting electrodes into deep regions of the brain.	A strong magnetic field is generated by placing an electromagnetic coil above the scalp, which acts on the cerebral cortex through the skull, inducing electrical currents and activating neurons.	By placing electrodes on the scalp, the cerebral cortex is stimulated by low-intensity direct current, thus regulating neural activity.
Advantage	Effective for deep brain areas, long-lasting effects.	Precise localization, non-invasive, and high safety.	Simple to use, non-invasive, high safety, low cost.
Disadvantage	Requires surgery, expensive equipment, potential side effects.	Limited effect on deep brain areas, may cause headaches.	Weaker stimulation effect, and variable outcomes due to individual differences.

With regards to combination therapy, exploring the integration of DBS with other therapeutic modalities, such as pharmacological therapy or cognitive intervention, is certainly feasible and may enhance overall treatment outcomes for patients with AD. Previous studies have investigated the combination of DBS with medication for AD (Zhang et al., 2021; Jiang et al., 2022); however, these studies did not compare the effects of DBS combined with medication to that of DBS alone or medication alone. More studies are required to assess the impact of combining DBS with other therapies on AD treatment outcomes.

## Conclusion

The options for AD therapy are as diverse and complex as its causes. Currently, there is a lack of targeted, effective, and safe therapeutic approaches for AD. It is becoming increasingly vital to develop comprehensive therapies that are not limited to a single method or solely reliant on pharmaceutical intervention. Recent studies conducted by researchers in the field of DBS have demonstrated its potential as a novel and promising non-pharmaceutical therapeutic approach for AD patients, supported by both clinical and basic research.

**Figure 4:**
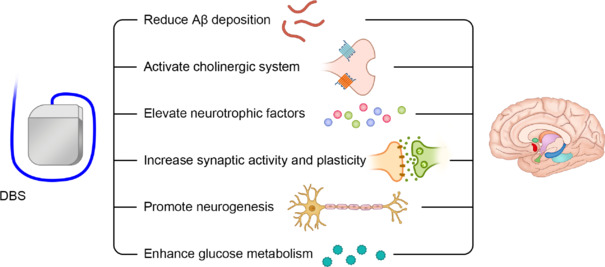
Mechanisms of DBS in AD therapy. DBS may exert therapeutic effects on AD through various mechanisms, including reducing Aβ deposition, activating the cholinergic system, elevating neurotrophic factors, increasing synaptic activity and plasticity, promoting neurogenesis, and enhancing glucose metabolism. AD: Alzheimer’s disease; Aβ: amyloid-beta; DBS: deep brain stimulation.

DBS may exert therapeutic effects on AD through various mechanisms, including reducing Aβ deposition, activating the cholinergic system, increasing neurotrophic factors, enhancing synaptic plasticity, promoting neurogenesis, and improving glucose metabolism (**Figure 4**). However, clinical trials investigating DBS for AD remain insufficient, and future efforts should focus on translating preclinical findings into clinical applications. Long-term follow-up studies are also necessary to evaluate the efficacy and safety of DBS treatment for AD, particularly regarding its impact on cognitive function, neuropsychiatric symptoms, quality of life, and changes in AD biomarkers. Previous studies on DBS for treating AD have often lacked sufficiently large sample sizes, and the selection of stimulation sites remains unoptimized while stimulation parameters are not standardized. More studies are needed to explore the effects of different DBS patterns on clinical outcomes. In this review, we comprehensively summarize the latest research findings on DBS, covering all known targets. We systematically review the progress regarding efficacy, underlying mechanisms, and limitations of DBS in both clinical and basic studies for treating AD. We also describe the anatomy and function of each stimulation target in detail and compare different neuromodulation techniques. This review aims to deepen the understanding of DBS in AD therapy and to guide further research. In the future, we should focus on multi-center clinical trials of DBS with large sample sizes, particularly within earlier therapeutic windows, such as the prodromal and even preclinical stages of AD, by which more effective and safer DBS therapies for patients with AD are explored.

## Data Availability

*Not applicable*.
